# HOW NETWORKS OF MEDICARE BENEFICIARY PATIENT TRANSFERS AFFECT HOSPITAL C. DIFFICILE OUTBREAK RISK

**DOI:** 10.1093/geroni/igz038.1801

**Published:** 2019-11-08

**Authors:** Catherine C Cohen, Timothy Luke Muggy

**Affiliations:** RAND Corporation, Santa Monica, California, United States

## Abstract

Clostridium difficile (CDI) is the most common healthcare associated infection (HAI), and it causes particularly high morbidity and mortality among older adults. While risk factors for this deadly HAI have been explored at the patient and facility levels, less is known about transmission between facilities. Recent literature suggests that more patient transfers to a hospital are correlated with elevated CDI levels. However, this work excludes skilled nursing facilities (SNFs), although SNFs are a major contributor of older adult patients to hospitals. We use multiple large data sets to make progress in filling several existing knowledge gaps. First, we construct and analyze healthcare facility networks using transfers of Medicare beneficiaries in the Minimum Data Set 3.0 and Medicare claims data from New Mexico, New York, Connecticut, and Colorado. We evaluate the role of SNFs in networks through the volume and frequency of patient flows in and out of individual hospitals. We also assess the level of interaction (i.e., transfers) between facilities of different sizes, locations, and types. Second, using the Healthcare Cost and Utilization Project State Inpatient Databases, we examine whether a hospital CDI outbreak is predictive of CDI incidence across a network of hospitals, using multiple existing metrics of CDI incidence, noting strengths and weaknesses for each. These two aims lay the foundation for future work to examine the relationship between patient transfers and the distribution of outbreaks. Such work may be able to identify facilities that present the greatest CDI risk to older adults across a facility network.

